# Acoustic trapping of active matter

**DOI:** 10.1038/ncomms10694

**Published:** 2016-03-10

**Authors:** Sho C. Takatori, Raf De Dier, Jan Vermant, John F. Brady

**Affiliations:** 1 Division of Chemistry and Chemical Engineering, California Institute of Technology, Pasadena, California 91125, USA; 2 Department of Materials, ETH Zürich, Vladimir-Prelog-Weg 5, Zürich 8093, Switzerland; 3 Department of Chemical Engineering, KU Leuven, Leuven B-3001, Belgium

## Abstract

Confinement of living microorganisms and self-propelled particles by an external trap provides a means of analysing the motion and behaviour of active systems. Developing a tweezer with a trapping radius large compared with the swimmers' size and run length has been an experimental challenge, as standard optical traps are too weak. Here we report the novel use of an acoustic tweezer to confine self-propelled particles in two dimensions over distances large compared with the swimmers' run length. We develop a near-harmonic trap to demonstrate the crossover from weak confinement, where the probability density is Boltzmann-like, to strong confinement, where the density is peaked along the perimeter. At high concentrations the swimmers crystallize into a close-packed structure, which subsequently ‘explodes' as a travelling wave when the tweezer is turned off. The swimmers' confined motion provides a measurement of the swim pressure, a unique mechanical pressure exerted by self-propelled bodies.

## Abstract (web summary)

Active matter, such as swimming bacteria, show unique behaviors under confinement, but it is experimentally challenging to measure them. Takatori *et al*. show the use of acoustic tweezers to trap self-propelled Janus particles as an enabling tool to investigate collective motions in living systems.

## Graphical abstract

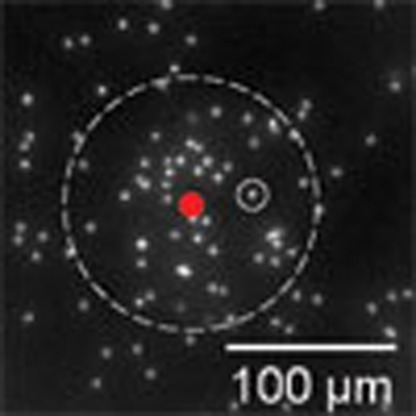

The study of active matter systems such as swimming bacteria and molecular motors in geometrically confined environments plays an essential role in understanding many cellular and biophysical processes. Many studies have demonstrated that self-propelled bodies exhibit intriguing phenomena in confined spaces, such as accumulation at boundaries^1^,^2^,^3^,^4^,^5^. In addition to confinement by a physical boundary, confinement of active particles via a harmonic external field can engender many useful properties of active systems^6^,^7^,^8^. However, this has remained an experimental challenge because most biological trapping instruments are too weak to confine active systems over a large spatial extent, as swimmers' run lengths can easily exceed 100 μm.

In this work, we overcome this challenge by developing a custom-built acoustic tweezer to confine self-propelled Janus particles in a two-dimensional (2D) near-harmonic trap. Analogous to optical or magnetic tweezers, acoustic traps employ sound waves to move objects to special regions of the acoustic radiation field. One-dimensional or 2D standing wave devices^9^,^10^ utilize axial radiation forces of multiple transducers and/or reflectors to manipulate objects into pressure nodes or antinodes. Unlike studies that impose a strong trap to manipulate structures and place objects at specified locations, our goal is to interrogate the motion and behaviour of the trap constituents themselves (that is, active microswimmers) under confinement.

Invoking the transverse acoustic forces of a single-beam transducer^11^, in the present work we use a 2D device capable of confining active particles over a wide range of trapping forces and spatial extents of the trap, from larger than the run length of the swimmers to much smaller than it. In addition to having a significantly stronger trapping force compared with optical tweezers, acoustic traps preclude potential laser damage to biological specimens (‘opticution')^12^ especially for the high power intensities required for our study. Our judicious choice of the trap strength enables us to study the non-equilibrium behaviours of active particles in varying degrees of confinement: for weak traps, the probability density is Boltzmann-like and can be mapped to that of classical equilibrium Brownian systems, and for strong traps, the density is peaked along the trap perimeter. We discover that the external trap behaves as an ‘osmotic barrier' confining swimmers inside the trapping region, analogous to semipermeable membranes that confine passive Brownian solutes inside a boundary. From the swimmers restricted motion inside the trap, we calculate the unique swim pressure generated by active systems originating from the mechanical force required to confine them by boundaries. Finally, we investigate the crossover from ballistic to diffusive behaviour of active microswimmers by observing the ‘explosion' of an active crystal.

## Results

### Active Janus particles in acoustic confinement

We fabricate Janus particles (platinum/polystyrene) that swim near the interface in hydrogen peroxide solution via self-diffusiophoresis^13^,^14^. These active Brownian spheres translate with an intrinsic swim velocity *U*_0_, tumble with a reorientation time 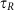, and experience a hydrodynamic drag *ζ* from the surrounding continuous Newtonian fluid. The tumbling of the swimmer from rotational Brownian motion results in a random walk process for 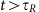 with translational diffusivity 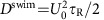 in 2D (Swimmers' translational Brownian diffusivity 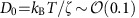 μm^2^ s^−1^ is small compared with 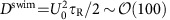
μm^2^ s^−1^, and *D*_0_ is not considered here.). We choose active synthetic Janus particles as a model living system with narrow distributions in swim velocity and reorientation time, but our tweezer setup can accommodate bacteria and other biological microswimmers.

Our acoustic trap exerts a Gaussian trap force^11^ with spring constant *k* and width *w*, *F*^trap^(*r*)=−*kr* exp(−2(*r*/*w*)^2^), which is well-approximated by a harmonic trap *F*^trap^(*r*)≈−*kr* for small departures 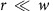. The Janus particles explore the interior of the trap with their intrinsic swimming motion; however, they are confined to remain within a circular boundary because they cannot travel beyond a certain distance from the trap centre where the magnitude of their self-propulsive force equals that of the trapping force, *F*^trap^(*r*)=*F*^swim^≡*ζU*_0_; *F*^swim^ is the swimmers' propulsive force that can be interpreted as the force necessary to hold the swimmer fixed in space. For a harmonic trap, the swimmers are confined within a radius *R*_*c*_≡*ζU*_0_/*k* of the trap centre. We measure the positions and mean-square displacement (MSD) of the swimmers in both weak and strong confinement as they explore the trapping region (see Methods section for further experimental detail). We also conduct Brownian dynamics computer simulations to corroborate the experimental measurements (see Methods section for simulation detail).

### Probability distribution

**Figure 1:**
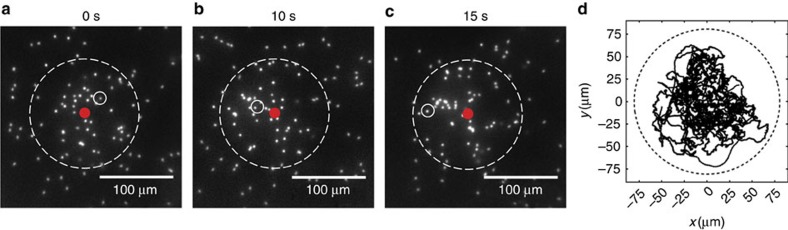
Active Janus particles in a weak acoustic trap. (**a**–**c**) Snapshots of 2 μm swimmers in an acoustic trap. The solid red spot indicates the trap centre and the dashed white circle delineates the outer edge of the well. The swimmer shown inside the solid white circle undergoes active Brownian motion while exploring the confines of the trap. (**d**) Two-dimensional trajectories of several particles inside the trap.

**Figure 2:**
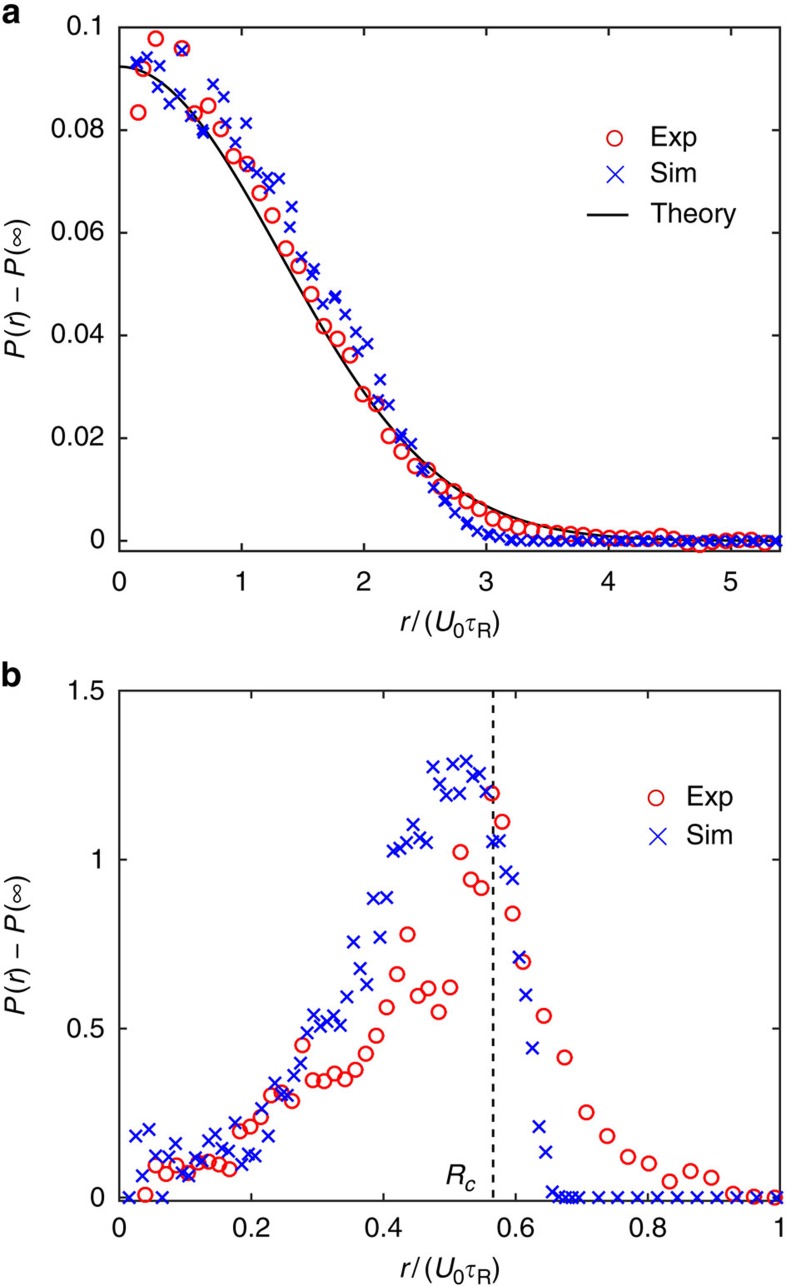
Probability distribution of confined active Janus particles. (**a**) 2 μm swimmers with 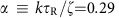 follow a Boltzmann distribution (solid black curve is the analytical theory, equation 1). (**b**) Distribution of 3 μm swimmers with *α*=1.76 has a peak near *R*_c_=*ζU*_0_/*k* (vertical dashed black line) and decreases to zero for *r*>*R*_c_. In both **a**,**b**, the red and blue symbols are data from experiment and Brownian dynamics simulations, respectively. Data are averages of measurements of over 500 snapshots for a duration of 50 s, each frame consisting ≈100 and 20 particles for the *α*=0.29 and *α*=1.76 cases, respectively.

A passive Brownian particle confined in a harmonic trap has the familiar Boltzmann probability distribution: *P*(*r*)∼exp(−*V*(*r*)/(*ζD*)), where *D* is the translational diffusivity. Since the active Brownian motion of a swimmer can be interpreted as a random walk, the distribution of swimmers in a trap is also Boltzmann with the swim diffusivity 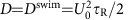:


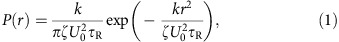


or by non-dimensionalizing, 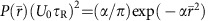, where 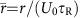 and 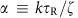 is the nondimensional trap stiffness that dictates the swimmer behaviour inside the trap. For a weak trap, *α*<1, the swimmers are allowed to explore and reorient freely before reaching the ‘ends' of the well (Fig. 1); the maximum density occurs at the trap centre *r*=0. Equation 1 is valid for *α*≲1 since the swimmers must be allowed to undergo a random walk process within the confines of the well^6^,^8^. As shown in Fig. 2a, equation 1 agrees with both experiments and Brownian dynamics simulations. The uniform density far away from the trap, *P*(∞), has been subtracted in the experiments. The active Janus particles have a range of activity levels due to variations in the platinum coating during fabrication. With a weak trap, strong swimmers are able to swim straight past the trap without getting confined, whereas the weaker swimmers struggle to escape the vicinity of the trap centre. The swimmer properties (speed *U*_0_ and reorientation time 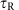) in equation 1 are the average of those particles that are confined within the trapping region.

In the other limit of a strong trap, *α*>1, the swimmer sees the ‘ends' of the well before it is able to reorient (that is, 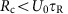), so the swimmer will be stuck at *R*_c_ until it reorients and then run quickly to the other side and again wait there^7^. As shown in Fig. 2b we observe a peak in the probability distribution near *R*_c_=*ζU*_0_/*k*, and the Boltzmann distribution no longer applies. To observe Brownian-like motion the spring must be weak, that is, 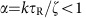, so that the particle can undergo a random walk before it discovers the ends of the well. Along the trap perimeter for large *α*, the particles are on average oriented radially outward relative to the trap centre. This directional alignment is most pronounced near *r*=*R*_c_—the particles want to swim away but the trapping force confines them to remain inside. The correlation between the distance from the trap centre and the particles' orientation is directly related to the mechanical swim pressure exerted by these particles (see later subsection). Videos of active particles in confinement are available in Supplementary Movies 1, 2, 3.

### Explosion of a ‘swimmer-crystal'

**Figure 3:**
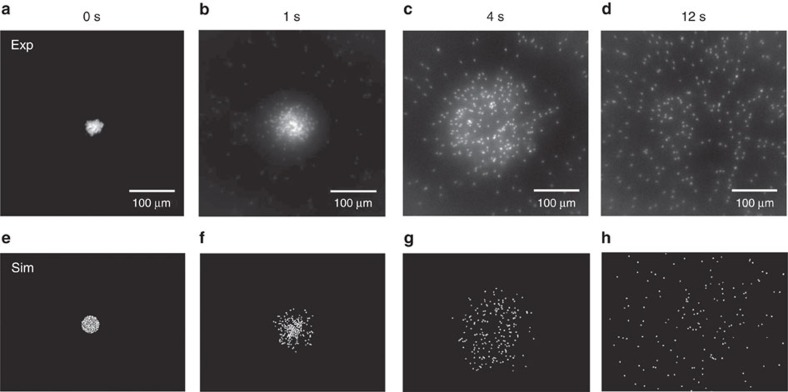
‘Explosion' of active crystal. Explosion of swimmer crystal in (**a**–**d**) experiments and (**e**–**h**) Brownian dynamics simulations. (**a**,**e**) A strong trapping force draws the swimmers into a dense close-packed 2D crystal. (**b**,**f**) A subsequent release of the trap frees the swimmers, causing the crystal to explode. (**c**,**g**) At later times, a ballistic shock propagates outward like a travelling wave. (**d**,**h**) At long times, the swimmers spread diffusively.

We have focused thus far on a dilute concentration of swimmers subjected to a relatively weak trap. Using a stronger trap, all swimmers that wander within the trapping region (∼150 μm radius from trap centre) are pulled towards the trap centre and form a dense close-packed 2D crystal (see Fig. 3a,e).

When the trap is subsequently turned off, the crystal quickly ‘melts' or ‘explodes' and the constituent particles swim away (see progression in Fig. 3). Videos of the accumulation, crystal formation, and melting process are available in Supplementary Movies 4, 5, 6. On first glance, this process resembles the melting of an active crystal due to the constituents' sudden loss of motility^15^. Palacci *et al*.^15^ use polymer/hematite particles that self-propel and interact with each other via long-ranged phoretic attraction in the presence of blue–violet light. Due to concentration–field interaction, the particles cluster and form crystals in the presence of light. When light is shut off, the crystal melts because the particles' motility and concentration–field interactions are turned off, and the now-passive particles spread with their translational Brownian diffusivity—the entire melting process is diffusive. In contrast, our active crystal explodes due to a sudden loss of an external trap forcing the particles together, not the slow diffusion process caused by a loss of swimmer motility. Thus, the motion of the spreading swimmers is still that of active particles—translating with speed *U*_0_ in randomly oriented directions that relax with the reorientation timescale 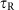.

We observe three time regimes in the explosion process. For times very short after release, only the swimmers positioned along the periphery of the crystal, escape the crystal. Particles in the centre obstruct each others's paths and are unable to escape the crystal, so the density is peaked at the origin (Fig. 3b,f). During the second regime the escaped particles move ballistically outwards in the direction given by their random initial orientation. The swimmers move ballistically because they have not yet reoriented sufficiently to be diffusive (that is, times 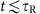). The result is a depletion of particles from the origin (given the initial crystal is small; see below) and a peak in the density that propagates outward like a travelling wave (Fig. 3c,g). Finally, for times 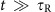 the swimmers have reoriented sufficiently to behave diffusively (Fig. 3d,h) characterized by the translational diffusivity *D*=*D*_0_+*D*^swim^ where *D*_0_ is the Stokes–Einstein–Sutherland diffusivity and 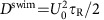. In this regime, the spreading process is analogous to the classic diffusion of an instantaneous point source, where the transient probability distribution is


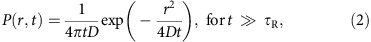


where *t* is the time after the point source is introduced and *D* is the translational diffusivity of the constituent ‘solute' particles. For the swimmer crystal, the diffusivity of the constituent particles 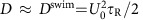.

**Figure 4:**
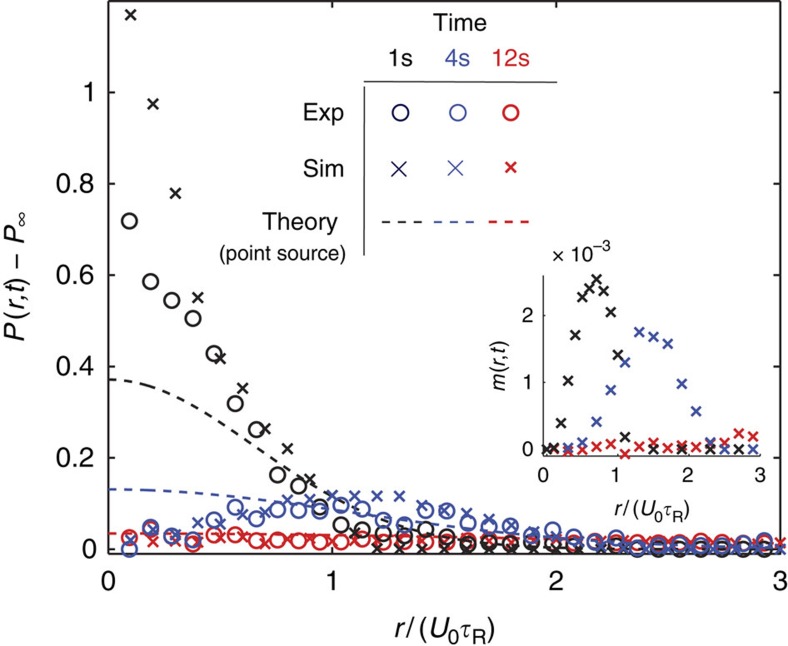
Evolution of active crystal ‘explosion'. Transient probability density of 2 μm swimmers as they explode from the crystal, drawn at three representative times as described in the text. Dashed curves are the analytical theory of diffusion of a point source, equation 2 (for 1 s and 4 s, drawn as a reference for comparison), and the circles and crosses are the experiment and Brownian dynamics simulation, respectively. Inset shows the polar order of the swimmers *m* (*r*, *t*) as the peaks spread outward. We average over four independent explosion measurements for a duration of 30 s after release; each run consists of ≈150 spreading particles.

Figure 4 corroborates our observations that show the three distinct time regimes in experiments and Brownian dynamics simulations. At short times, particles inside the crystal spread slightly faster in the experiment than the simulation, perhaps due to the sudden release of a strong acoustic force causing the particles to relax and loosen the initial packing of the crystal (see 1 s in Fig. 4). As predicted, equation 2 is valid only for times 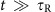 when the explosion process is diffusive (shown as dashed curves in Fig. 4 for comparison). This is a distinguishing feature of our explosion experiments compared with the melting of a passive Brownian crystal^15^. Because the swimmers' reorientation time can be large compared with the measurement times (as opposed to Brownian momentum relaxation times that are small), we are able to observe the crossover between ballistic and diffusive explosion of the active crystal. There is no ballistic regime for the melting of passive Brownian particles.

We conduct this experiment with different particle activities and initial crystal sizes. When the initial crystal is large and/or the swimmers reorient rapidly (that is, 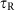 is small), the ballistic shock and depletion of swimmers from the crystal origin was less noticeable. For a large initial crystal, the particles in the centre must wait long times to break free of the cluster; by this time, swimmers initially positioned along the crystal periphery have had enough time to reorient and explore back towards the origin to fill the void.

Inside the crystal there is an effective interaction of the concentration fields due to the swimmers' competition for ‘fuel' (hydrogen peroxide). However, for a system that can be assumed to be a 2D monolayer, fuel is being supplied from the third dimension (that is, the bulk) and thus the competition is reduced. Data near the trap centre are difficult to analyse in the experiments (especially at short time), since we cannot accurately differentiate between individual particles in the large crystal. Due to the finite size of the initial crystal, theoretical prediction for diffusion of a step function (as opposed to a delta function) would appear to be more accurate at very short times after release, but equation 2 is valid for 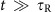, so there is little difference between the two solutions in this regime.

This experiment provides a macroscopic method to measure the diffusivity of an active system using tweezers. The size or width of the spreading swimmer crystal is related to the diffusivity by *L*^2^=2*Dt*, so one can measure the size of the spreading system (ignoring details about the motion of individual swimmers) for times 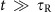 to infer the diffusivity from 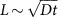.

These ‘explosion' experiments show the development of local polar order of the swimmers as they spread outward. Orientational polar order is established when the swimmers' motion is directionally aligned. Inset of Fig. 4 shows the average swimming orientation 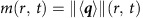 at three representative times, where ***q***=(cos *θ*, sin *θ*) is a unit orientation vector defined by the swimmer's direction of self-propulsion (see Methods section for further detail), and the brackets 〈·〉 indicate an average over all particles at a distance *r* from the crystal centre. One interpretation of *m*(*r*, *t*) is the local average orientation distribution, 〈cos(*θ*′)〉, where *θ*′ is the angle of the swimmer orientation relative to the outward normal from the crystal centre. Local polar order is peaked along the perimeter of the crystal, and spreads radially outward with time like a wave front. For short times when the system is ballistic, there is coherent motion of particles in the outward radial direction. This directed behaviour cannot be seen for a purely passive Brownian system, which exhibits ‘biased-diffusion' for all times. At longer times when the crystal melts completely, there is no polar order.

Finally, a possible interpretation of the explosion process is that the particles are spreading from regions of large mechanical pressure (centre of crystal) to small mechanical pressure (far away from crystal). Spatial gradients in the active mechanical pressure results in an outward flux of constituent particles^16^—in the next section we compute the unique mechanical swim pressure exerted by active systems.

### Swim pressure

In addition to the probability density and diffusivity of confined swimmers, the acoustic trap can be used as a measurement of the swim pressure^16^, a unique mechanical pressure that all self-propelled bodies exert as a result of their self-motion. The origin of the swim pressure is that all active bodies exert a mechanical, self-propulsive force on the surrounding boundaries that confine them per unit area, Π^swim^=*F*^wall^/*A*. A swimmer's self-propulsive force is given by ***F***^swim^≡*ζ**U***_0_ where *ζ* is the drag factor and ***U***_0_ is the swimming velocity. This is the force required to hold the swimmer fixed, at every instance in time. The swim pressure differs from the swim force, and also differs from the random thermal Brownian osmotic pressure due to the different intrinsic timescale. An active particle that collides into a wall will not reorient as a result of the collision—it continues to ‘push' against the wall until it finally reorients from its intrinsic reorientation mechanism. These continuous collisions of magnitude *F*^swim^ against the wall over the time duration 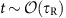 is what gives rise to the swim pressure.

Although many theoretical studies^16^,^17^,^18^,^19^,^20^,^21^ have analysed the swim pressure of active matter, there is a dearth of experimental corroboration. A recent study on sedimentation^22^ gives an indirect measurement based on density profiles of active particles under gravity. Because the acoustic trap behaves as an invisible ‘semipermeable membrane' that confines the swimmers (but allows the solvent to pass through), our experiment allows us to determine the swim pressure using principles analogous to the osmotic pressure of colloidal solutes in solution.

We use the virial theorem^23^ to express the swim pressure as a force moment^16^: 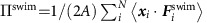 in 2D where *N* is the number of particles, *A* is the system area, and the swim forces of each particle 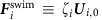. As shown in the Methods section, the first moment of the equation of motion for an active particle gives 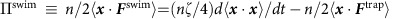, where *n* is the number density of swimmers. In the absence of the trap, ***F***^trap^=0, we have Π^swim^=*nζD*^swim^, where we have expressed the first term on the right using the diffusivity, *D*^swim^=(1/4)*d*〈***x***·***x***〉/*dt*. Here the swimmer undergoes an entropic, random walk in unbounded space with 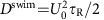, giving the ‘ideal-gas' swim pressure 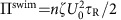 (ref. 16). In the presence of a trap force, ***F***^trap^, the long-time diffusivity *D*^swim^=0 because the swimmers are confined and cannot translate in unbounded space, and the MSD achieves a constant value (that is, does not grow with time). Therefore, at steady-state *d*〈***x***·***x***〉/*dt*=0 and the acoustic trap acts as a confining force that is equal and opposite to the swim force, enabling us to determine the swim pressure via the known trap force: Π^swim^=−*n*/2〈***x***·***F***^trap^〉.

For a harmonic trap where ***F***^trap^=−*k**x***, the swim pressure can be obtained directly by measuring the MSD of the swimmer inside the trap:


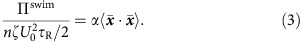


where 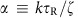 and 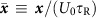 is the nondimensional position vector of the swimmer relative to the trap centre. This elegant result reveals that the MSD contains information about the mechanical pressure exerted by self-propelled particles. It comes directly from the virial theorem, where the force moment becomes a MSD for a harmonic trap.

Solving the Langevin equation analytically for a swimmer confined in a trap (see Methods section), we obtain


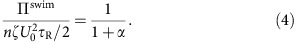


The swim pressure depends only on the parameter 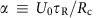, a ratio of the swimmers' run length 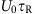 to the size of the trap *R*_c_=*ζU*_0_/*k*. Therefore, this expression gives the container-size dependent swim pressure—for a weak trap, *α*→0, and we obtain the ‘ideal-gas' swim pressure 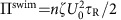, whereas a strong trap causes the fictitious ‘container' to shrink and decrease Π^swim^ because the distance the swimmers travel between reorientations decreases. Because the swim pressure is a force moment, the distance the swimmers travel between reorientations (that is, within a time 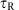) is the ‘moment arm' that determines the magnitude of the swim pressure. For a weak trap the particles are allowed to travel the full run length 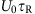, set by the particles' intrinsic swimming speed *U*_0_, whereas a strong trap establishes a small trap size 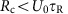 that obstructs and causes the particles to travel a distance smaller than 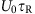.

**Figure 5:**
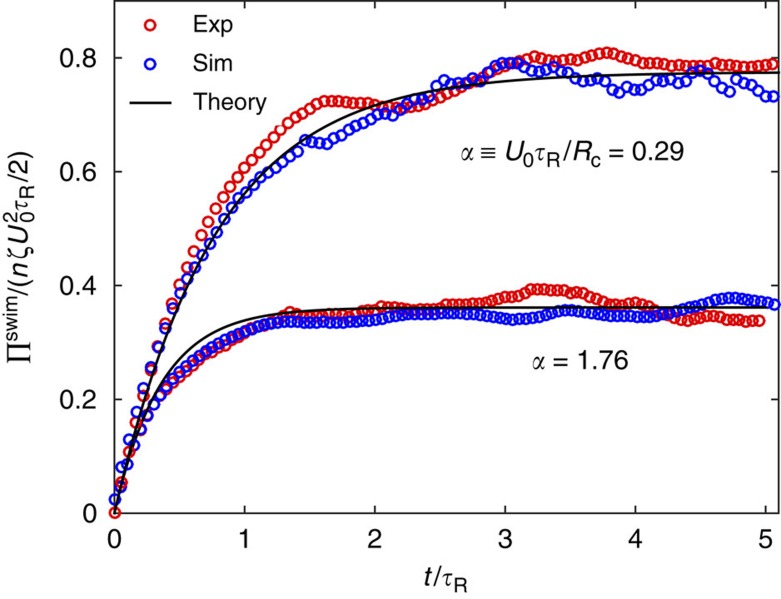
Swim pressure of Janus particles in different degrees of confinement. The parameter 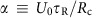 is a ratio of the particles' run length to the trap size *R*_c_=*ζU*_0_/*k*. The solid black curves are the theoretical prediction with a harmonic trap approximation, and the red and blue symbols are results from experiments and Brownian dynamics simulations, respectively. A smaller trap size diminishes the distance the particles travel between reorientations and decreases the swim pressure. The experimental and simulation data are averages of 150 and 90 independent particle trajectories for a duration of 40*s* for the *α*=0.29 and *α*=1.76 cases, respectively.

Figure 5 shows the swim pressure computed in the experiments and Brownian dynamics simulations using equation 3. The swim pressure has transient behaviour for small times and we require data for times 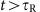 to observe a steady state (Methods section). At steady state, all curves approach the theoretical prediction given by equation 4.

As a swimmer wanders away from the trap centre, it may explore regions of the trap that are not strictly in the linear Hookean regime. Therefore one may expect the MSD to be slightly higher than the linear theory. However, although the swimmer concentration away from the trap is dilute, near the trap centre swimmers accumulate and cluster, which obstructs the motion of free swimmers trying to swim across to the other end of the trap, decreasing the MSD. Hydrodynamic interactions may also play a role near the trap centre where the density of swimmers is higher. The analytical theory is valid for a dilute system of swimmers in a linear harmonic trap without hydrodynamic interactions, but we find that the linear approximations are sufficient. In addition to the MSD and equation 3 (which come from a linear approximation of *F*^trap^), we also compute the full correlation using a Gaussian *F*^trap^ (Methods section) and the results have minor quantitative differences.

We scale the swim pressure in Fig. 5 using the average activity of the swimmers confined within the trapping region. The number density *n* is given by the number of trapped particles *N* divided by the area of the trapping region.

When a weak trap is present for a long time (≳20–30 min) there is a gradual accumulation of swimmers inside the trap because those located initially far away wander near the trap and become confined. This induces a slow variation in the number density inside the trap *n*(*t*) over time. We use a dilute system of swimmers (total area fraction *φ*_*A*_≤0.001) and the timescale for the change in number density (≥5 min) is large compared with the swimmers' reorientation time (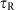
∼2–10*s*). Since the important timescale in our problem is the swimmers' reorientation time 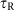, we only require data over a timespan of several 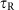 and the effect of swimmer accumulation is negligible in our results.

## Discussion

We do not observe a self-pumping state^7^ induced by hydrodynamic interactions, perhaps due to the dilute concentrations of this study. Although we do not observe large-scale coherent motion, the precise manipulation of swimmers towards special regions may provide a method to study the collective motion of living systems in a controlled manner. Further experiments using acoustic traps may give insight into the origin of polar order, how and why living organisms align, and the advantages of such collective behaviour.

Our measurement of the swim pressure may support forthcoming applications in biophysics and molecular cell biology, as researchers are becoming increasingly concerned with the mechanical forces, pressures, and stresses generated by active constituents inside a living cell. In addition, experimental determination of the swim pressure may engender real-life engineering applications, such as fabrication of novel soft materials using active swimmers.

## Methods

### Equations of motion

The Langevin equation for a dilute system of swimmers in a trap is given by


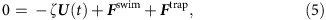


where ***U***(*t*) is the velocity, *ζ* is the hydrodynamic drag factor, ***F***^*swim*^≡*ζ**U***_0_ is the self-propulsive swim force of a swimmer, ***U***_0_=*U*_0_***q*** is the intrinsic swim velocity of an isolated swimmer, ***q***(*t*) is the swimmer's unit orientation vector defined by their swimming direction, and ***F***^trap^=−∇*V*(***x***) is the restoring force caused by the trap with potential *V*(***x***). The left-hand side of equation 5 is zero because inertia is negligible for a colloidal dispersion.

Transverse trapping with an acoustic tweezer results in a Gaussian trap^11^ with stiffness *k* and width *w*, 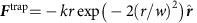, which we independently verify. We use Janus particles in the absence of hydrogen peroxide (that is, inactive particles) to calibrate *k* and *w* of the acoustic trap by measuring the position and velocity of the particles in the trap. For a trap with large spatial extent (large *w*), a linear force 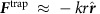 approximates the trapping force. As a swimmer wanders far away from the focus of the trap, there is a critical radius ∼*R*_c_=*ζU*_0_/*k* at which the swimmer cannot move any farther. At this position the swimmer's self-propulsive force ***F***^swim^ exactly cancels the trapping force ***F***^trap^ and the swimmer does not move. The swimmer is ‘stuck' in this position for a time of order 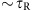 until the swimmer changes its orientation. The trapping force does not affect particles located far away from the trap origin because ***F***^trap^(***r***→∞)→**0**.

### Experimental methods

We fabricate active Janus particles from 2 and 3 μm diameter sulfate latex particles (Life Technologies, Carlsbad, CA, USA). We coat half of the particle surface with a ∼7-nm thick layer of platinum using a BAL-TEC SCD 050 sputter coater (Leica Microsystems GmbH, Wetzlar, Germany). When deposited in a hydrogen peroxide solution, the particles self-propel via diffusiophoresis near the air-water interface. The particles initially deposited into the bulk rise towards the air/solution interface because the platinum half is heavy and orients with gravity. The particles swim towards the non-platinum face, and begin to move in 2D once they reach the interface. The particles at the interface do not diffuse back into the third dimension (the bulk). For the 2 and 3 μm particles, they have a swim speed of *U*_0_∼15–25 μm s^−1^ and ∼8–15 μm s^−1^ with a reorientation time of 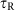
∼2–5 s and ∼5–10 s, respectively. We compute the reorientation time by analysing the swimmers' orientation autocorrelation: 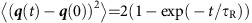. We verify that the swimmers undergo active Brownian motion characterized by the swim diffusivity 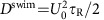.

To confine the swimmers in the transverse direction we develop a custom-built acoustic tweezer setup. We excite a 0.25-inch diameter immersion type transducer (UTX Inc., Holmes, NY, USA) in a continuous sinusoidal signal at 25 MHz with variable voltages from 0 to 10*V*_pp_ using an AM300 Dual Arbitrary Generator (Rohde & Schwarz, Munich, Germany). We immerse the transducer in the solution in an inverted position (face up to the air/solution interface) and deposit Janus particles on this interface. We adjust the focal point of the transducer at a distance of 12 mm from the air/solution interface and hold it fixed in place throughout the experiment using an XY positioner and a tilt stage (Thorlabs Inc, Newton, NJ, USA). Although the transducer also has a radiation force in the axial direction, the effect of particles in the bulk being pushed to the interface is negligible because the Janus particles remain on the interface and do not diffuse in three dimension into the bulk. We control the strength of the trap by changing the input voltage from the function generator. We connect a × 50 objective (Leica Microsystems GmbH, Wetzlar, Germany) to a sCMOS digital camera (ORCA-Flash 4.0, Hamamatsu, Japan) to obtain images and a glass fibre ringlight (Volpi AG, Schlieren, Switzerland) to provide lighting. Most commonly researchers use acoustic tweezers to generate a standing field to confine objects primarily in one dimension in acoustic pressure nodes or antinodes, depending on the properties of the objects (density, compressibility)^9^. Here we develop a 2D device which generates a near-harmonic potential using the transverse radiation forces of single-beam transducer.

We deposit Janus particles on the air-water interface of a 0.5 wt% hydrogen peroxide solution, and their activity remains constant for at least 1 h (each experimental run last ∼ few minutes). We turn on the acoustic transducer and observe the motion of the swimmers in confinement within the trapping region. For the small voltages we apply to the transducer (0–3*V*_pp_) and the particle sizes (2 and 3 μm) used in our swim pressure measurements, we do not detect any acoustic streaming. The acoustic tweezer exerts a Gaussian trapping force on the particles; a linear Hookean spring force approximates the trapping force since the width *w* is large compared with the swimmers' run lengths. We identify the centre of the trap at the end of each experiment by applying a strong trapping force to collect all of the swimmers to the trap centre. We use a modified particle tracking script^24^ in the analysis.

### Brownian dynamics simulations

In our Brownian dynamics simulations we evolve the particles following equation 5. Although the concentration of swimmers far away from the trap centre is dilute, we may have an accumulation of swimmers near the trap centre that may obstruct the motion of free swimmers trying to swim across to the other end of the trap. To more accurately model the experimental system, our Brownian dynamics simulations include the interparticle force ***F***^P^ in equation 5. Nondimensionalizing the force by *ζU*_0_ and position by 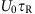, equation 5 (with the interparticle force) becomes 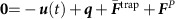, where ***u***≡***U***/*U*_0_ is the particle velocity, 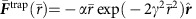 is the trapping force, 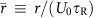 is the radial position of the swimmer, 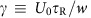 is a ratio of the swimmers' run length to the trap width, and 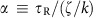 is a ratio of the swimmers' reorientation time to the timescale of the trap. We can also interpret 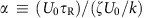, the ratio of the swimmers' run length to the ‘size' of the container (set by the trap). For Figs 2 and 5 we use *α*=0.29, *γ*=0.08 and *α*=1.76, *γ*=0.25 for a weak and strong trap, respectively. We vary the number of particles from 20 to 500 to match the experimental measurements. We use a hard-disk interparticle force ***F***^P^=***F***^HS^ that prevents particle overlap in our simulations^25^,^26^. We evolve the swimming orientation of the swimmers ***q***=(cos *θ*, sin *θ*) following 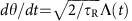 where Λ(*t*) is a unit random deviate.

### Derivation of the swim pressure in a harmonic trap

For a harmonic trapping force ***F***^trap^=−*k**x***, we can solve equation 5 for the position ***x***(*t*) and compute the MSD:


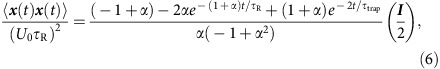


where ***I*** is the isotropic tensor and *τ*_trap_=*ζ*/*k* is the characteristic timescale of the trap. For small times the MSD grows quadratically in time, and for *α*=0 we obtain the long-time self diffusivity of an active swimmer: 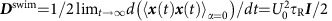. Most importantly, for *α*≠0 and times long compared with both 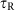 and 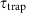 the MSD becomes a constant


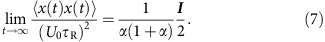


This is a main result that we will use later.

Multiplying equation 5 by *n**x*** and taking the average we obtain


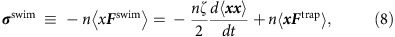


where we use the definition of the swim stress 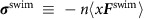 and *n* is the number density of swimmers^16^. As shown in equation 7, for times long compared to both 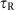 and 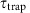 the MSD becomes a constant and its time derivative is zero: 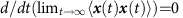. Therefore, the swim pressure Π^swim^=−tr***σ***^swim^/2 (in 2D) is


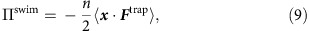


which is a general result valid in principle for any trapping force ***F***^trap^. For a harmonic trap ***F***^trap^=−*k**x***, the swim pressure can be determined from a simple MSD measurement as given in equation 3 of the main text. Substituting equation 7 into equation 3, we obtain the theoretical result 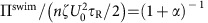 as given in equation 4 of the main text.

For times not large compared to 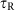 and 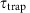, the slope of MSD is not zero and the swim pressure has a transient start up period:


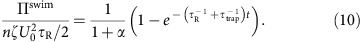


This expression is exact and valid for all times *t*. On taking times 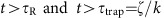, this result agrees with equation 4. Therefore, measuring the MSD 〈***xx***〉 is an easy and simple method to quantify the swim pressure in an experimental system.

For nonlinear traps with a general form of ***F***^trap^, we must evaluate equation 9 directly. For a Gaussian trap with stiffness *k* and width *w*, 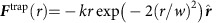, we have


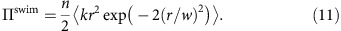


For a large well (large *w*), the trapping force becomes harmonic and we get back the previous result in equation 3, where the MSD 〈*r*^2^〉 gives the swim pressure.

## Additional information

**How to cite this article:** Takatori, S. C. *et al*. Acoustic trapping of active matter. *Nat. Commun.* 7:10694 doi: 10.1038/ncomms10694 (2016).

## Supplementary Material

Supplementary Movie 1 Experiment 2μ*m* of swimmers in a weak acoustic trap. The trap focus is at the center of the video, and the swimmers far away from the focus are unaffected by the trap. The swimmers undergo active Brownian motion while exploring the confines of the well. Near the end of the video we turn off the acoustic transducer and the swimmers immediately spread out. We conduct repeat measurements to gather sufficient statistics.

Supplementary Movie 2 Experiment of 2μ*m* swimmers at higher concentrations in a weak acoustic trap. The video is sped up by 4 times. Particle tracking is difficult at higher concentrations so we conduct repeat measurements at low concentrations to gather sufficient statistics.

Supplementary Movie 3Brownian dynamics (BD) simulation of trapped swimmers inside a weak acoustic trap, with nondimensional spring constant 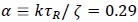 and trap width 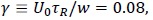, where 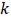 is the trap stiffness, 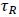 is the swimmer reorientation time, 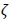 is the hydrodynamic drag factor, 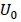 is the swim speed, and *w* is the trap width.

Supplementary Movie 4Experiment of 2μ*m* swimmers forming an 'active crystal' using a strong acoustic trap, and subsequently 'exploding' when the trap is turned off. The particles have an average swim speed 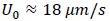 and reorientation time 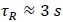. We observe the crossover from ballistic to diffusive explosion of the active crystal. In contrast, there is no ballistic regime for the melting of passive Brownian particles.

Supplementary Movie 5Experiment of 3μ*m* swimmers exploding from the active crystal when the acoustic transducer is turned off. The particles have an average swim speed 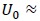 12μ*m/s* and reorientation time 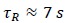.

Supplementary Movie 6Brownian dynamics (BD) simulation of the active crystal exploding due to an imbalance of the active mechanical pressure. Initially, the particles were placed at random close-packed positions. This video shows 200 particles with a nondimensional parameter 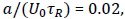 where α is the particle size and 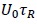 is the run length of the swimmer.
